# Impact on Knowledge, Competence, and Performance of a Faculty-Led Web-Based Educational Activity for Type 2 Diabetes and Obesity: Questionnaire Study Among Health Care Professionals and Analysis of Anonymized Patient Records

**DOI:** 10.2196/49115

**Published:** 2023-09-13

**Authors:** Jennifer Okemah, Sola Neunie, Alexander Noble, Carol Wysham

**Affiliations:** 1 Salute Nutrition PLLC Kirkland, WA United States; 2 touch Independent Medical Education Stockport, Cheshire United Kingdom; 3 Rockwood Center for Diabetes and Endocrinology Spokane, WA United States

**Keywords:** continuing medical education, incretin-based therapy, multidisciplinary care, obesity, overweight, type 2 diabetes, patient data, diabetes management, patient education, chronic disease, web-based education, digital education, health education, diabetes, diabetes mellitus, survey, web-based survey, education, glycemic

## Abstract

**Background:**

Strategies for managing type 2 diabetes (T2D) and obesity are evolving with the introduction of targeted therapies, including incretin-based dual agonists and growing knowledge of the importance of multidisciplinary care. Accessible, effective continuing medical education (CME) activities are required to ensure that health care professionals (HCPs) understand and can implement the most recent data to optimize patient outcomes.

**Objective:**

We aimed to measure changes in knowledge, competence, and self-reported performance and quantitatively evaluate changes in performance using anonymized patient data following participation in a web-based educational activity. The faculty-led CME-accredited activity was based on incretin-based dual agonists and patient education on T2D and obesity. The remaining educational gaps in this field were also identified.

**Methods:**

A CME-accredited, web-based, multidisciplinary (touchMDT) educational activity titled “The future for glycemic control and weight loss in T2D and obesity: Incretin-based dual-agonists and optimizing patient education” was developed. HCP knowledge, competence, and performance were assessed before and after the activity against Moore’s expanded outcomes framework (levels 1-5), using self-reported questionnaires and by analyzing anonymized patient record data.

**Results:**

For evaluating knowledge and competence (50 respondents before and 50 learners after the activity), the mean number of correctly answered questions was significantly higher post activity (median 5.0, IQR 4.0-6.0 to 6.0, IQR 5.0-7.0; mean 4.98, SD 1.22 to 5.78, SD 1.13; *P*<.001). Modest, nonsignificant improvements in self-reported performance (N=50 respondents preactivity; N=50 learners postactivity) from before to after the activity were observed (median 4.0, IQR 3.25-4.0 to 4.0, IQR 4.0-4.0; mean 3.64, SD 0.69 to 3.76, SD 0.48; *P*=.32). PPatient data analysis indicated that patients were being treated more intensively postactivity: before the activity, the most commonly used treatment regimens were metformin monotherapy (13/50, 26%) and dual therapy with metformin plus injectable glucagon-like peptide-1 (GLP-1) receptor agonist (RA; 11/50, 22%); post activity, this changed to dual therapy with metformin plus injectable GLP-1 RA (12/50, 24%) and triple therapy with metformin plus injectable GLP-1 RA plus sodium-glucose cotransporter-2 inhibitor (SGLT2i; 10/50, 20%). In addition, there was an increased number of referrals to a combination of specialists (physicians referred 27%, 8/30 of patients to ≥2 specialists before the activity and 36%, 10/28 to ≥2 specialists post activity). The remaining educational gaps included understanding the biology and psychology of obesity, efficacy and safety data for incretin-based dual agonists, and the role of the diabetes educator or diabetes care and education specialist in managing T2D and obesity.

**Conclusions:**

This short, web-based CME activity on the management of T2D and obesity led to improvements in HCP knowledge, competence, and performance. Several remaining unmet needs were identified, which can be used to inform the content of future educational activities in this disease area.

## Introduction

### Burden of Type 2 Diabetes and Obesity

The prevalence of diabetes has reached epidemic proportions globally, with the International Diabetes Federation estimating that in 2021, more than half a billion adults (537 million) will be living with the condition [1]. Type 2 diabetes (T2D) accounts for over 90% of all cases of diabetes worldwide and is strongly associated with overweight and obesity [1]. Obesity contributes to the etiopathogenesis of T2D and is associated with the development of T2D-associated complications [2]. To optimize outcomes, both obesity and T2D must be effectively managed together; for example, weight loss in patients with T2D improves insulin sensitivity and long-term outcomes [3].

Obesity rates are rising rapidly, and it is estimated that 1 in 5 women and 1 in 7 men will be living with obesity (defined as a BMI ≥30 kg/m^2^) by 2030 [4]. The treatment of obesity remains challenging with lifestyle modifications, such as calorie restriction and increased physical activity often not resulting in sustained weight loss when used as a stand-alone treatment [5]. Thus, effective pharmacotherapies that target the underlying pathophysiology of metabolic diseases, such as T2D and obesity, are an attractive treatment option and have been the focus of much research in recent decades.

### Incretin-Based Therapies

Incretin hormones, such as glucose-dependent insulinotropic polypeptide (GIP) and glucagon-like peptide-1 (GLP-1), are gut peptides that are released in response to oral nutrient intake [6]. In healthy individuals, incretins have multiple functions, including inhibition of glucagon secretion, stimulation of glucose-dependent insulin secretion, decreased gastric emptying, and reduced food intake [7,8]. Over the past 2 decades, several incretin-based therapies, including GLP-1 receptor agonists (RAs) and dipeptidyl peptidase-4 (DPP-4) inhibitors, have been developed and approved for the treatment of both T2D and obesity. The GLP-1 RAs directly stimulate GLP-1 receptors in the pancreas and brain, leading to improved glycemic control and reduced appetite. Exenatide was the first GLP-1 RA to be approved by the US Food and Drug Administration as a treatment for T2D in 2005. Subsequently, liraglutide became the first GLP-1 RA to be approved as a treatment for both T2D (2010) and obesity (2014) [5]. The DPP-4 inhibitors prevent the breakdown and inactivation of GLP-1 and GIP, thus increasing their plasma half-lives. In 2006, sitagliptin became the first DPP-4 inhibitor to be approved for the treatment of T2D [9]. Since their initial development, the pharmacokinetic profile of GLP-1 RAs has been improved through various chemical modifications. This has led to the development of twice-daily, daily, and weekly subcutaneous formulations, as well as an oral formulation of semaglutide [5].

Emerging evidence has demonstrated that coinfusion of GLP-1 and GIP exerts a synergistic effect in healthy individuals [10], and new dual agonists have shown promising results in clinical trials [11-13]. For example, the dual GIP and GLP-1 RA tirzepatide at 5-15 mg was shown to be superior to basal insulin and to 1 mg of semaglutide weekly in reducing levels of hemoglobin A_1c_ (HbA_1c_) and body weight in patients with overweight or obesity and T2D [11,12]. Subsequently, tirzepatide was approved by the US Food and Drug Administration for the treatment of T2D in 2022 and was described as having very high efficacy for reduction in HbA_1c_ and body weight in the 2022 American Diabetes Association and the European Association for the Study of Diabetes consensus statement [14].

### Unmet Need for Patient and Health Care Professional Education in T2D and Obesity

The American Diabetes Association and the European Association for the Study of Diabetes guidelines recommend clinicians reassess and modify treatment after 3-6 months if HbA_1c_ levels consistently stay above target [14]. However, real-world data have shown that initial treatment intensification typically does not occur for over a year after first recognizing failure in achieving glycemic targets [15,16]. The reasons behind this delay remain to be fully elucidated; however, health literacy may be a barrier for patients in understanding the value of therapy intensification in T2D management [17]. There is also a need to improve training in weight management in T2D. Despite the urgent need for weight loss, a survey found that fewer than half (49%) of nurses and primary care physicians reported receiving specialty training in weight management since their initial medical training, yet 79% of them reported being interested in education on patient-directed weight management strategies [18], emphasizing the need for education on guideline-based disease management.

The importance of responsive and effective health care professional (HCP) education was highlighted by a position statement published by the Insights for Diabetes Excellence, Access, and Learning Group in 2020 to meet the increasing needs for diversity, specialization, cultural competence, advancing practice, and person-centeredness in diabetes care delivery [19]. Patients with T2D and diabetes can benefit from a coordinated approach with a dedicated multidisciplinary team (MDT) focused on weight loss, dietary advice, managing symptoms and side effects, and dosing of medications. The MDT may include a consultant endocrinologist and diabesity specialist nurse, a specialist dietitian, an exercise physiologist, and a psychologist [20,21]. Diabetes care and education specialists (DCESs; previously known as certified diabetes educators) also play a key role in the MDT model [22]. The value of DCESs to patient outcomes was shown in a recent meta-analysis that demonstrated greater improvements in HbA_1c_ following nurse- or DCES-based interventions compared with physician-based interventions [23].

### Optimal Format of HCP Education

For HCPs with busy clinical schedules, it can be difficult to find time to attend traditional face-to-face continuing medical education (CME) activities [24,25]. As an alternative, web-based activities have become increasingly popular, particularly in response to the limitations imposed by the COVID-19 pandemic [26]. Web-based activities are accessible to HCPs, irrespective of their location and training budget, and they can also be developed and viewed at a time convenient to both the expert faculty and learners [25]. Short-duration educational activities that are succinct and easy to digest have also recently become an important part of CME [27].

### Objectives and Aims of This Analysis

In this study, we developed and implemented a faculty-led, CME-accredited, web-based educational activity on the future role of incretin-based dual agonists and the importance of patient education in T2D and obesity. The objectives of this analysis were (1) to measure changes in knowledge, competence, and self-reported performance after participation; (2) to objectively evaluate changes in learners’ performance using anonymized patient data; and (3) to identify remaining educational gaps in this field.

## Methods

### Educational Activity

Educational gaps and learning objectives were identified and formulated by touch Independent Medical Education, an organization that provides independent medical education for HCPs, through a review of the published literature and feedback from expert faculty specializing in the treatment of T2D and obesity.

A faculty-led, web-based, multidisciplinary (touchMDT) activity was developed by touch Independent Medical Education in collaboration with the faculty and was free to access on-demand on the touchENDOCRINOLOGY website [28] from March 28, 2022, to March 28, 2023. The activity comprised three 10-15–minute videos (providing 37 minutes of education in total) and involved MDT members (an endocrinologist specializing in diabetes, an endocrinologist specializing in obesity, and a DCES) discussing their role in the management of patients with T2D and obesity with a patient with T2D.

The target audience was endocrinologists based in the United States involved in the management of patients with T2D and obesity. CME accreditation was provided by the University of South Florida Health, which is accredited by the Accreditation Council for Continuing Medical Education as a provider of continuing professional development. Details of the activity and the learning objectives are provided in Multimedia Appendix 1. Communication channels used to reach the target audience are included in Multimedia Appendix 1.

### Assessment of Educational Outcomes

Outcomes for both educational activities were assessed using a comprehensive system of analysis, in line with the 7-level framework for assessing the outcomes of CME programs developed by Moore et al [29] in 2009. For this study, Moore’s levels 1 to 5 were assessed (participation, satisfaction, knowledge, competence, and performance).

#### Levels 1 and 2—Participation and Satisfaction

Level 1 (participation) included the number of HCPs who engaged in the activity and the average time spent viewing the video. Level 2 (satisfaction) was assessed using a postactivity questionnaire. Full details are provided in Multimedia Appendix 1.

#### Levels 3 to 4—Knowledge and Competence and Level 5—Self-Reported Performance

**Figure 1:**
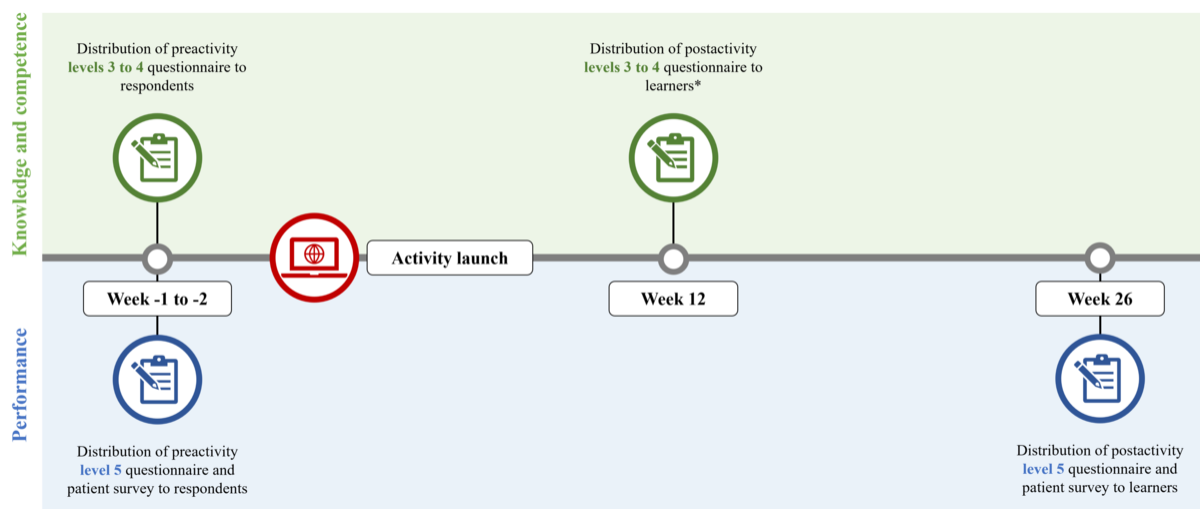
Questionnaire distribution.

Levels 3 to 4 and level 5 (self-reported) were assessed using outcome questionnaires, with all data collected by an independent third party (nuaxia Limited) that, to avoid bias, was not involved in the development of the activities. The target audience for the outcome questionnaires was predefined as endocrinologists based in the United States to ensure the sample was taken from relevant respondents (HCPs who completed the preactivity questionnaire) and learners (HCPs who participated in the activity and completed the postactivity questionnaire). To avoid any pre-exposure bias and to obtain a statistically representative sample size, data were collected using an independent sample model both before and after launch of each activity. Questionnaire distribution and timing are shown in Figure 1, in which all outcome questionnaires were fielded to a database of 6453 HCPs and closed once a prespecified number had responded. The level 3 to 4 preactivity questionnaire was fielded 1-2 weeks before launch (to ensure the sample was from HCPs who had not interacted with the activity), and the postactivity questionnaire was fielded to another set of HCPs 12 weeks after launch. The level 5 questionnaire and the first patient record digital questionnaire were fielded 1-2 weeks before launch—to a different set of HCPs than those who answered the level 3 to 4 questionnaires. Twenty-six weeks after launch, the level 5 questionnaire and the second patient record digital questionnaire were administered to the same HCPs who completed the level 5 questionnaire before launch. The asterisk denotes that level 2 (satisfaction) was assessed as part of the postactivity levels 3 to 4 questionnaire.

The level 3 to level 4 and level 5 questionnaires comprised 7 and 4 questions, respectively. All questions were developed by touch Independent Medical Education Medical Directors and approved for medical accuracy by the faculty. Level 3 to level 4 questions were multiple-choice and included 4 possible answers, of which only 1 was correct. Level 5 questions were multiple-choice with 3 to 4 plausible answers, of which 1 was highlighted as the best clinical option. An overview of the topics included in the level 3 to level 4 and level 5 questionnaires is shown in Textbox 1. Full questionnaires are included in Multimedia Appendices 2 and 3.

**Topics included in the level 3 to 4 and level 5 outcomes questionnaires.**

**Levels 3 and 4**

- Action of endogenous incretin hormones
- Mechanism of action of the dual glucose-dependent insulinotropic polypeptide (GIP) and glucagon-like peptide-1 (GLP-1) receptor agonist tirzepatide
- Potential benefits of incretin-based dual GIP and GLP-1 RAs for patients with type 2 diabetes (T2D) and obesity
- Efficacy of a dual GLP-1 and glucagon receptor agonist (cotadutide) in patients with T2D and overweight
- Rationale for early intensification of therapy beyond metformin
- Use of add-on therapies in patients with established atherosclerotic cardiovascular disease (ASCVD)
- Management of patients with difficulties using injection device

**Level 5**

- Second-line treatment options for patient with poorly controlled T2D and obesity
- Side effects associated with second-line treatment with a GLP-1 RA
- Management of patients on dual therapy but with hemoglobin A_1c_ above their individual target
- Patient education on T2D disease management and potential complications

#### Level 5—Patient Data–Based Performance

Changes in performance were also assessed through an evaluation of anonymized patient data. HCPs were sent a digital questionnaire to complete on 2 separate occasions: 1-2 weeks before and 26 weeks after the activity. This included 16 questions that captured data from two key fields: (1) general patient demographics and history and (2) early treatment intensification and weight loss strategies. Each HCP was requested to extract all relevant information from a single patient record into a digital questionnaire, ensuring no personal or identifiable patient information was disclosed. As many participants who engaged in the preactivity test were included in the postactivity test as possible; however, due to the dropout rate over the time between data collection, additional HCPs needed to be questioned postactivity. Participant specialties were validated by artificial intelligence–aided examination of data from several sources, including hospitals, professional publications, and prescribing and insurance databases. HCPs were asked to confirm that they treat the relevant condition.

### Self-Reported Confidence and Intent to Change Practice

As part of the level 3 to level 5 questionnaires, respondents and learners were asked, “How confident are you in treating T2D and obesity?” (level 3 to level 4 questionnaire only), and learners were asked, “As a result of your participation in this session, will you make a change in your practice?” Mutually exclusive responses to the confidence question were not confident, a little confident, somewhat confident, moderately confident, and extremely confident; and to the change in practice question, they were yes, uncertain—more education needed, uncertain—practical limitations, no—more education needed, and no—practical limitations.

### Identification of Outstanding Educational Gaps

Four potential educational gaps were included in the level 3 to level 5 questionnaires, and participants were asked to rank them by importance. The results were analyzed by specialty using a Single Transferable Vote system as described previously [30]. Educational gaps were also identified through an analysis of responses to the questionnaires assessing level 3 to level 5 outcomes. Questions that were answered incorrectly by ≥30% of learners after completion of the educational activity were identified as outstanding educational gaps. The cutoff of 30% was based on an analysis of outcomes data from previous educational activities by touch Independent Medical Education.

### Statistical Analyses

The data were analyzed using SPSS Statistics (version 28.0.1; IBM Corp). Knowledge, competence, and self-reported performance (levels 3-5) outcomes were compared for the overall population using an independent sample *t* test and in subgroups defined by years of experience (<1-10 years, >10-20 years, and >20 years for levels 3-4; 0-20 and >20 years for level 5) using a 2-way ANOVA. Individual questions were analyzed with a paired sample *t* test followed by a 1-way ANOVA. For performance (level 5), as measured by anonymized patient data, a comparison between the pre- and postactivity data was made with a paired sample *t* test. The margin of error was 10%, based on the sample size of 50 for the pre- and postactivity data sets.

### Consent and Ethical Considerations

All consents and ethical confirmations were obtained within the questionnaire itself in accordance with British Healthcare Business Intelligence Association professional guidelines and General Data Protection Regulation consents. Where local markets have additional requirements, these were confirmed in line with the European Pharmaceutical Market Research Association’s professional guidance. This study did not report experiments on human participants; therefore, institutional review board approval and informed consent were not applicable.

## Results

### Assessment of Educational Outcomes

#### Levels 1 and 2—Participation and Satisfaction

By 12 months after launch, 8159 participants had engaged with the touchMDT activity, with an average participation time of 7 minutes. Overall satisfaction was 89%, with mean satisfaction scores (out of 5.0) of 4.4 for the quality of the activity, 4.5 for meeting learning objectives, 4.3 for being free from commercial bias, 4.4 for knowledgeable and effective presenters, 4.7 for relevance to clinical practice, and 4.3 for impact on management strategies.

#### Levels 3 and 4—Knowledge and Competence

**Figure 2:**
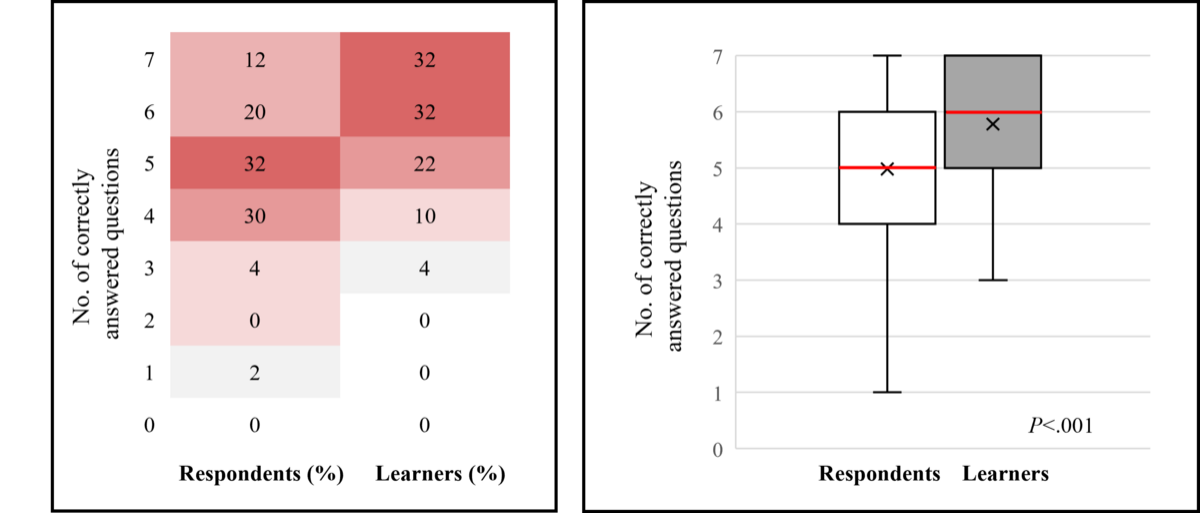
Summary of the number of correct responses for the level 3 to 4 outcomes questionnaires pre- and postactivity.

The level 3-4 questionnaire was completed by 50 respondents before and 50 learners after the activity. Before the activity, 32% (16/50) of respondents answered at least 6 questions correctly. This increased to 64% (32/50) of learners after the activity, as shown in Figure 2, where the heat map on the left shows the proportion of respondents (n=50) and learners (n=50) who answered specific numbers of questions correctly, as displayed by colors ranging from white (the lowest proportion of respondents and learners) to dark red (the highest proportion of respondents and learners). The box-and-whisker plot on the right shows the distribution of the number of correctly answered questions by all respondents and learners. The horizontal red line within the box indicates the median, the “x” symbol represents the mean, the boxes indicate the IQR, and the vertical lines (whiskers) extend to the range of values, excluding outliers. Outliers are defined as values that fall outside a distance of 1.5× the IQR from the upper and lower quartiles and are represented by empty circles. There was a statistically significant increase in the number of correctly answered questions from before to after the activity for all participants (median 5.0, IQR 4.0-6.0 to 6.0, IQR 5.0-7.0; mean 4.98, SD 1.22 to 5.78, SD 1.13; *P*<.001; Figure 2). A significant increase in the mean number of questions answered correctly from before to after the activity was observed in subgroups defined by years of experience (*P*=.01). The degree of improvement also differed by years of experience (*P*=.04; Multimedia Appendix 4).

**Figure 3:**
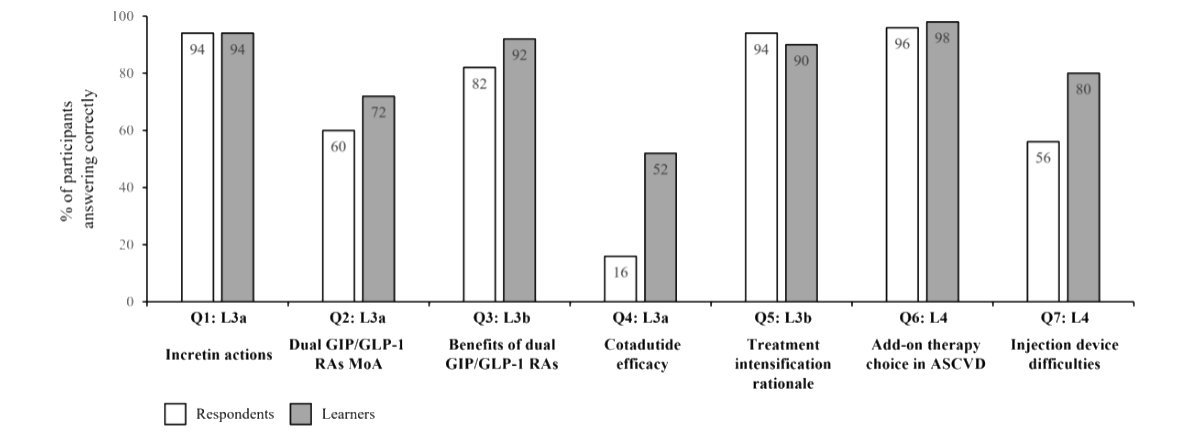
Summary of correct responses for individual topics for the level 3 to 4 outcomes questionnaire pre- and postactivity.

Before the activity, the question on the efficacy of the dual GLP-1/glucagon RA cotadutide for weight loss received the lowest number of correct answers (8/50, 16% of respondents). This increased to 52% (26/50) post activity, although it remained the question with the lowest number of correct answers. This is shown in Figure 3, where the bar graph shows the percentage of respondents (N=50) and learners (N=50) who answered each question correctly. Numbers within bars indicate their value. The magnitude of the change from before to after the activity was significantly different between the questions (*P*=.02).

#### Level 5

##### Self-Reported Performance

**Figure 4:**
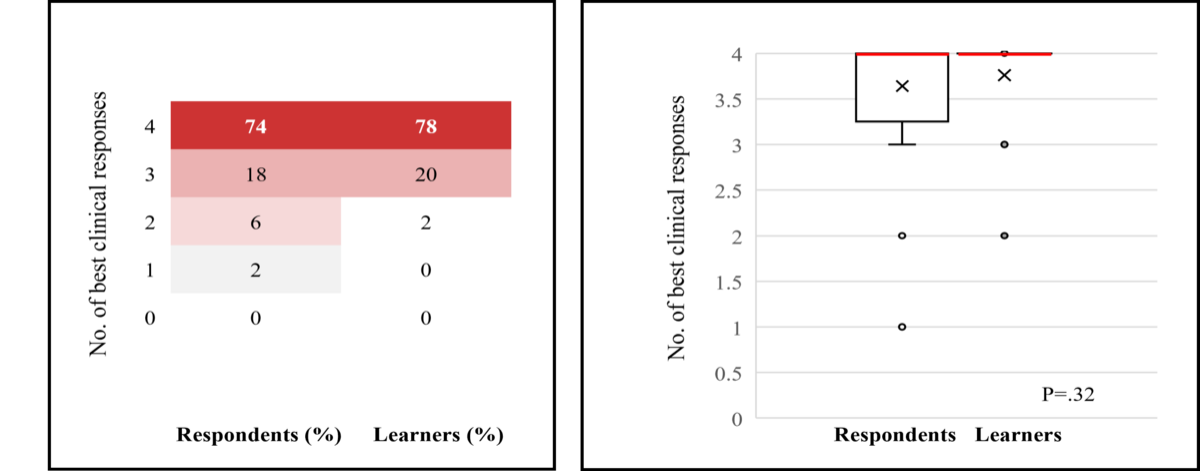
Summary of the number of correct responses for the level 5 outcomes questionnaires pre-and postactivity.

**Figure 5:**
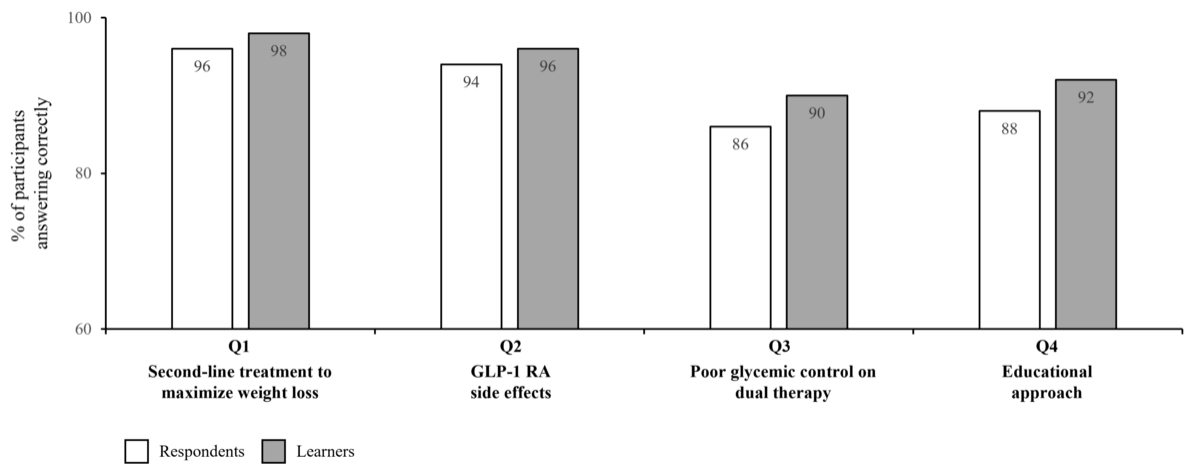
Summary of correct responses for individual topics for the level 5 outcomes questionnaire pre- and postactivity. GLP1: glucagon-like peptide-1; RA: receptor agonist.

Fifty respondents and learners completed the level 5 questionnaire. Before the activity, 74% (37/50) of respondents answered all 4 questions with the best clinical option, indicating a high level of baseline performance. This increased to 78% (39/50) of learners post activity and is shown in Figure 4; the heat map on the left shows the proportion of respondents (n=50) and learners (n=50) who answered specific numbers of questions correctly, as displayed by colors ranging from white (lowest proportion of respondents and learners) to dark red (highest proportion of respondents and learners). The box-and-whisker plot on the right shows the distribution of the number of correctly answered questions by all respondents and learners. The horizontal red line within the box indicates the median, the “x” symbol represents the mean, the boxes indicate the IQR, and the vertical lines (whiskers) extend to the range of values, excluding outliers. Outliers are defined as values that fall outside a distance of 1.5× the IQR from the upper and lower quartiles and are represented by empty circles. The increase in the number of correctly answered questions from before to after the activity for all participants was not statistically significant (median 4.0, IQR 3.25-4.0 to 4.0, IQR 4.0-4.0; mean 3.64, SD 0.69 to 3.76 SD 0.48; *P*=.32; Figure 4). The difference in best clinical option responses after the educational activity was not significant when analyzed by years of experience (*P*=.38; data not shown). Before the activity, the correct clinical option was selected by the majority of respondents for all questions (ranging from 86%, 43/50 to 96%, 48/50, as shown in Figure 5, where the bar graphs show the percentage of respondents [N=50] and learners [N=50] who answered each question correctly; the numbers within the bars indicate their value). Modest increases in the proportion of respondents providing the best clinical option from pre- to postactivity were recorded for all questions (2%-4% [an increase of 1-2 respondents out of 50]).

##### Patient Record–Based Performance

Fifty respondents and learners completed the level 5 patient records digital questionnaire. The dropout rate was 20% (n=10); therefore, 10 of the 50 learners were HCPs who were matched to the dropouts. Baseline patient characteristics reported by respondents and learners were broadly similar (Multimedia Appendix 5). Health care insurance types were variable for respondents and learners. Before the activity, the majority of patients had preferred provider organization health insurance (23/50, 46%), whereas after the activity, the most common health insurance types were preferred provider organization and health maintenance organization (18/50, 36% each).

**Figure 6:**
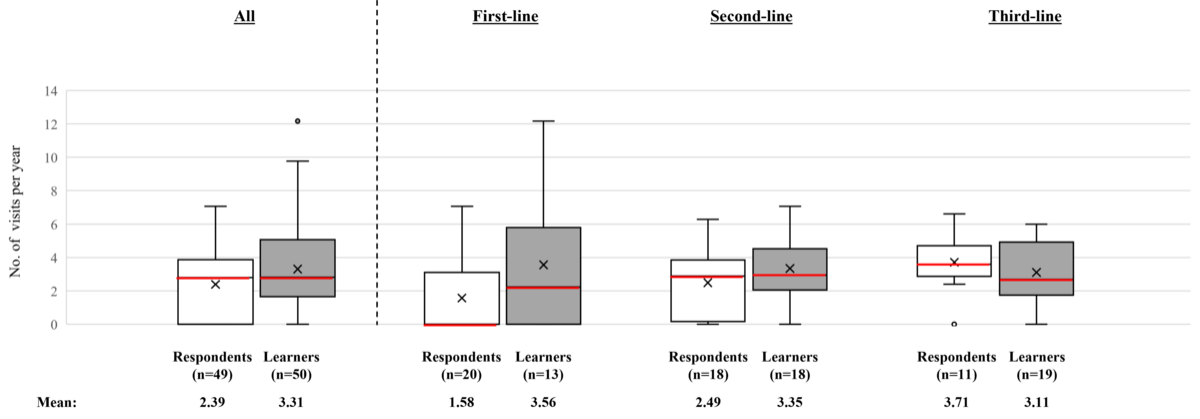
Number of patient visits reported by respondents and learners in the level 5 patient records questionnaire. GLP1: glucagon-like peptide-1; RA: receptor agonist; SGLT2i: sodium-glucose cotransporter-2 inhibitor.

**Figure 7:**
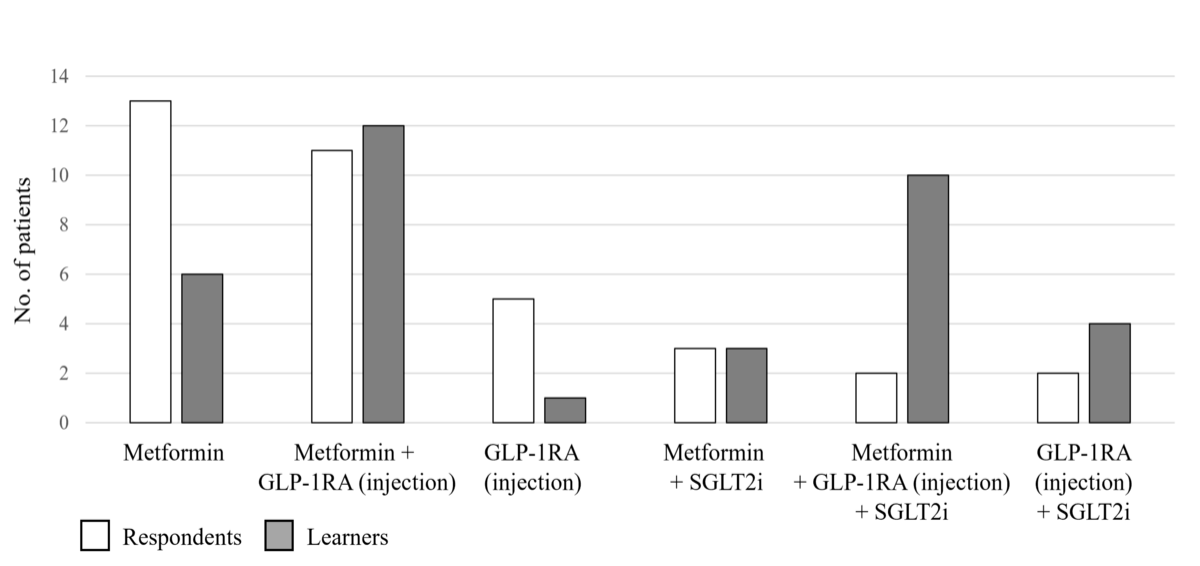
Diabetes treatment regimens reported by respondents and learners in the level 5 patient records questionnaire.

Before the activity, the mean number of patient visits per year increased in line with the number of lines of therapy: 1.58 for patients on first-line medications, 2.49 for patients on second-line, and 3.71 for patients on third-line. Following the educational activity, the number of patient visits per year increased by almost 1 (2.39 to 3.31; *P*=.06). The largest increase was for patients on first-line therapy (+1.98 visits per year). This is shown in Figure 6, where the box-and-whisker plot shows the distribution of the number of patient visits by all respondents and learners. The horizontal red line within the box indicates the median, the “x” symbol represents the mean (also stated below the plot), the boxes indicate the IQR, and the vertical lines (whiskers) extend to the range of values, excluding outliers. Outliers are defined as values that fall outside a distance of 1.5× the IQR from the upper and lower quartiles and are represented by empty circles. Data are shown for all patients and for patients in subgroups by first-, second-, and third-line therapy. Preactivity, the 2 most commonly used treatment regimens were metformin as monotherapy (13/50, 26%) or dual therapy with metformin and an injectable GLP-1 RA (11/50, 22%). After the activity, the most commonly used treatment regimens were dual therapy with metformin and an injectable GLP-1 RA (12/50, 24%) or triple therapy with metformin, an injectable GLP-1 RA, and a sodium-glucose cotransporter-2 inhibitor (SGLT2i; 20%, 10/50; Figure 7, where the bar graph shows the number of patients who received specific diabetes treatment regimens). The numbers within the bars indicate their value. Data are shown for all treatment regimens received by at least 3 patients (as reported by either the respondents or the learners). The proportion of patients who had a treatment added or switched at their most recent visit increased from 26% (13/50) before the activity to 30% (15/50) post activity. Of these, the majority added or switched to a GLP-1 RA (76.9%, 10/13 before the activity and 60%, 9/15 post activity; Multimedia Appendix 6). Before and after the activity, all physicians who added or switched to a GLP-1 RA did so to achieve weight loss, and the majority wished to improve the patient’s glycemic control, achieve cardiorenal benefits, or a combination of both (Multimedia Appendix 7).

**Figure 8:**
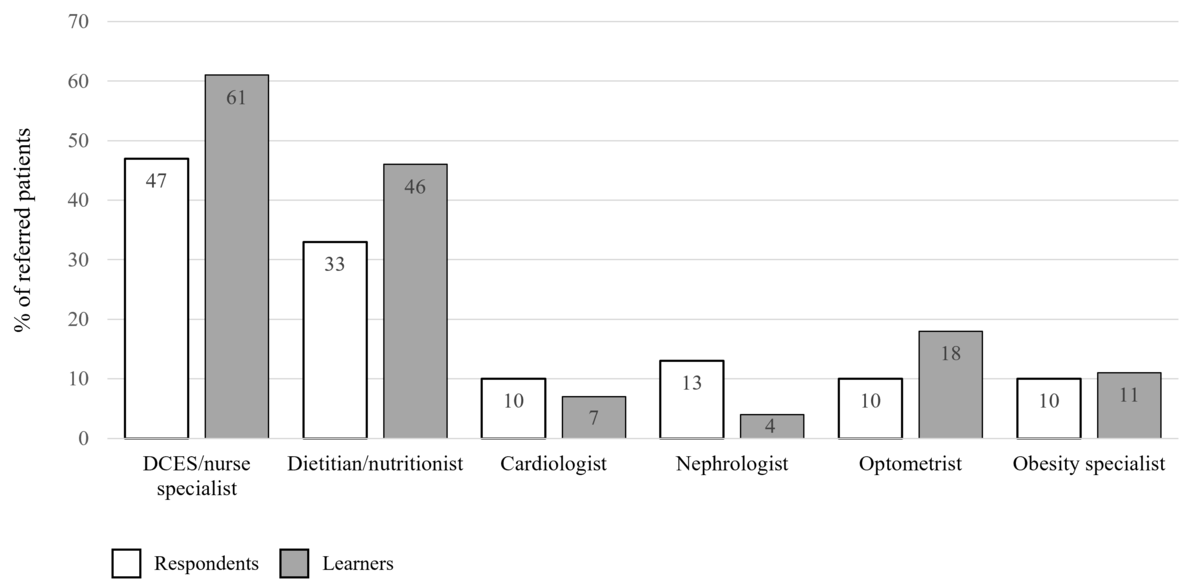
Specialist referrals reported by respondents and learners in the level 5 patient records questionnaire. DCES: diabetes care and education specialist.

**Figure 9:**
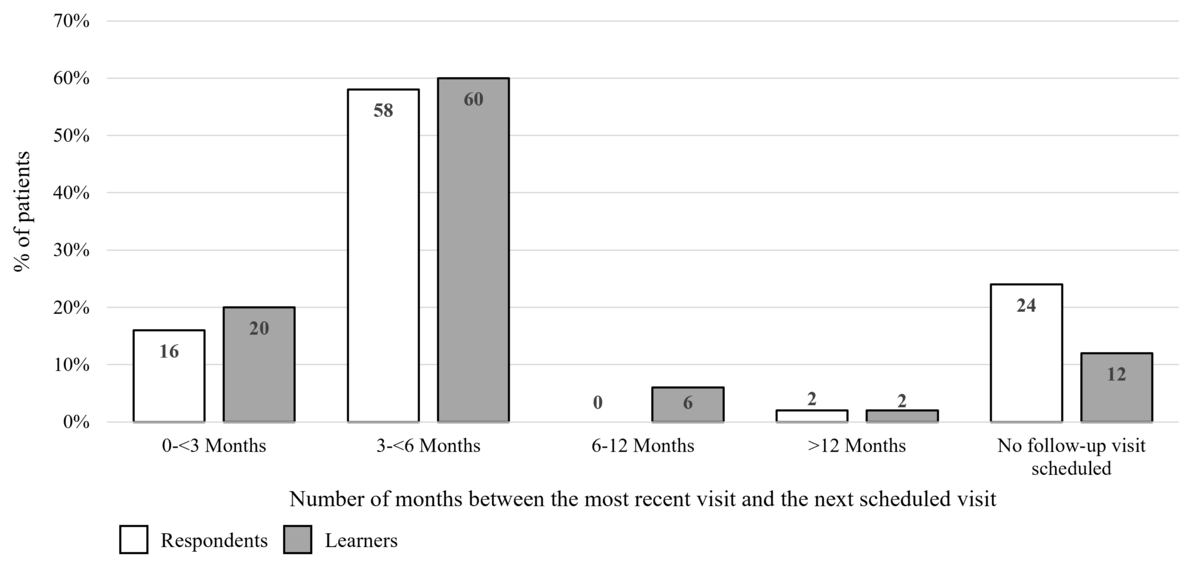
Follow-up scheduling reported by respondents and learners in the level 5 patient records questionnaire.

The proportion of patients who were referred to a specialist by physicians was similar before (30/50, 60%) and after (28/50, 56%) the activity (*P*=.69). Of these, among respondents, 14/30 (47%) patients had received a referral to a DCES or diabetes educator or a diabetes nurse specialist. This increased to 17 of 28 (61%) patients among learners. There was also an increase in the number of patients referred to a dietitian or nutritionist from before to after the activity (33%, 10/30 to 46%, 13/28). This is shown in Figure 8, where the bar graph shows the percentage of patients who received referrals to other specialists. The numbers within the bars indicate their value. DCESs can be multidisciplinary and may also be registered dietitians. For the purposes of this analysis, it was assumed that respondents and learners selected the primary specialty that they wished to refer the patient to. After the activity, physicians were also more likely to refer patients to a combination of specialists, with referrals to 2 or more specialists reported for 27% (8/30) of patients before the activity and 36% (10/28) of patients post activity. Before the activity, a follow-up visit was scheduled within 6 months by 74% (37/50) of physicians. This increased to 80% (40/50) after the educational activity. Furthermore, in the preactivity analysis, around a quarter of physicians (12/50, 24%) did not schedule a follow-up visit for their patients. This decreased to 12% (6/50) after the educational activity and is shown in Figure 9, where the bar graph shows the number of months between the most recent visit and the next scheduled visit for patients as reported by respondents and learners. The numbers within the bars indicate their value.

### Self-Reported Confidence and Intent to Change Practice

From responses to the level 3 to 4 questionnaire, there was an increase in confidence in treating T2D from before to after the activity, with the proportion of respondents reporting that they felt “extremely confident” increasing from 70% (35/50) to 84% (42/50). This was mainly driven by a decrease in those feeling “moderately” confident (24%, 12/50 to 10%, 5/50). Very few respondents described themselves as being “not confident” or “a little confident,” both before (0%) and after the activity (1/50, 2%). Overall, 62% (31/50) and 58% (29/50) of learners in the level 3 to 4 and level 5 outcomes questionnaires, respectively, stated that they would make a change to their practice following their participation in the educational activity (Multimedia Appendix 8).

### Identification of Outstanding Educational Gaps

In an analysis of answers to the postactivity level 3 to 4 questionnaire, 48% (24/50) of learners were unable to demonstrate procedural knowledge on the efficacy of cotadutide regarding weight loss in patients with T2D and overweight.

When asked what they considered to be their most important unmet educational needs, learners from the level 3 to 4 and level 5 questionnaires highlighted “understanding the biology and psychology of obesity and approaches for its prevention and management.” In addition, learners from the level 3 to 4 questionnaire highlighted “efficacy and safety data for incretin-based dual agonists and their future role in clinical practice,” and learners from the level 5 questionnaire highlighted “the role of the diabetes educator or DCES in the T2D and obesity multidisciplinary team” as their most important unmet educational needs (Multimedia Appendix 9).

## Discussion

### Principal Results

In this study, HCPs expressed high levels of satisfaction and improvements in their knowledge and competence in T2D and obesity management. Improvements in knowledge and competence were observed; modest improvements in self-reported performance were also recorded, although these were limited by the high levels of performance demonstrated before the activity.

Previous studies have shown that self-reported performance can be subjective and liable to bias (eg, recall bias or social desirability bias) [31,32]. Using a more objective method, such as analyzing patient records to assess changes in patient management, can help validate self-reported outcomes and provide insights into the impact of the educational activity on patient management. In this study, the analysis of anonymized patient data showed that patients were being treated more intensively after the educational activity. In particular, increases in the number of patient visits per year were observed, and there was a reduction in the number of patients without a follow-up visit scheduled. This suggests a reduction in clinical inertia, enabling prompt treatment intensification where required. In addition, there was an increased number of referrals to a combination of specialists, including diabetes educators or DCESs, diabetes nurse specialists, and nutritionists or dietitians in particular. This indicates that the educational activity may have been effective in communicating the importance of MDT care for patients with T2D and obesity. There was also a move away from monotherapy and an increase in patients receiving triple therapy with metformin, an injectable GLP-1 RA, and an SGLT2i. All physicians who added a GLP-1 RA to their patient’s treatment regimen stated that they did so with the aim of achieving weight loss, suggesting that the educational activity was effective in communicating the benefits of GLP-1 RAs beyond glycemic control. Taken together, these factors may explain why there was no overall increase in the volume of referrals after the educational activity**—**patients may have been achieving better overall outcomes due to the combination of increased visits, treatment intensification, and attention from a combination of specialists in the MDT. As such, the need for more specialist referrals was not warranted.

In agreement with the improvements in knowledge, competence, and performance, physicians reported increased confidence in the treatment of patients with T2D and obesity after participating in the educational activity. In addition, almost two-thirds stated that they would make a change to their practice. This is consistent with a previous study showing a correlation between improvements in confidence and a commitment to change clinical practice [33]. Practical limitations were cited as the reason why 25% of learners could not change their practice. This may reflect a lack of access to certain antihyperglycemic therapies, diabetes education channels, or both. Insights from another recent study on factors that impact HCPs’ intent to put newly acquired learning into practice suggest that the consequences of adopting new clinical behaviors and a lack of self-belief in one’s capabilities can also prevent HCPs from converting key learnings into tangible actions [34].

### Limitations

This study had several limitations:

1. Subgroup analyses were limited due to the small size of the subgroups.
2. All medical educational studies are affected by self-selection bias (ie, HCPs who feel they lack knowledge on a specific topic are more likely to participate).
3. Moore’s levels 6 and 7 and the long-term impact of the educational activities were not assessed.
4. The patient cohort included in the level 5 analysis was not completely reflective of the overall diabetes population in the United States. In 2018, the diabetes population was 57% White, 19% Hispanic, 15% Black, and 7% Asian [35]. In our cohort, Caucasian individuals were overrepresented, and therefore the results may not be completely generalizable to the overall US T2D population.
5. The term “DCES” was not strictly defined in the questionnaire, and the specific role was therefore open to interpretation by each respondent and learner.

### Comparison With Prior Work

The results presented here are consistent with a similar study that demonstrated that short, case-based, web-based CME led to improvements in knowledge, competence, and self-reported performance in T2D management [36]. Similarly, in another recent study, primary care physicians who participated in a tele-education program reported increased confidence in diabetes management and improvements in their ability to prescribe, manage, and troubleshoot diabetes technology [37].

### Identification of Needs for Further Education in T2D and Obesity

Participant feedback on educational activities is important to refine future educational activities, and several unmet educational needs were identified as part of this activity. Self-reported educational gaps were: “understanding the biology and psychology of obesity and approaches for its prevention and management,” suggesting that physicians are interested in the root causes of obesity and its prevention, not solely how to manage it; “efficacy and safety data for incretin-based dual agonists and their future role in clinical practice,” showing that physicians understand the potential impact of these agents and wish to be prepared to prescribe them to patients; and “the role of the diabetes educator or DCES in the T2D and obesity MDT,” showing that how diabetes educators or DCESs can be integrated into the MDT is not yet fully understood, but physicians are willing to learn more.

### Conclusions

This study demonstrated that a short-duration, free-to-access web-based CME activity on the management of T2D and obesity can lead to improvements in HCP knowledge, competence, and performance (both self-reported and as assessed by patient data). Several remaining unmet needs were identified, which can be used to inform the content of future educational activities in this disease area. Further studies will be valuable in defining the clinical impact of CME, particularly regarding the long-term impact of education on HCP performance and the benefits for patient and community health.

## Appendix

Multimedia Appendix 1 Supplementary methods.

Multimedia Appendix 2 Questions included in the level 3 and 4 outcomes questionnaire.

Multimedia Appendix 3 Questions included in the level 5 outcomes questionnaire.

Multimedia Appendix 4 Summary of correct responses for the level 3 to 4 outcomes questionnaire before and after the launch of touchMDT by level of experience of the respondents and learners.

Multimedia Appendix 5 Baseline characteristics for patients in the level 5 patient records questionnaire.

Multimedia Appendix 6 Additions or changes to diabetes treatment reported by respondents and learners in the level 5 patient records questionnaire.

Multimedia Appendix 7 Reasons given for addition or changes to diabetes treatment reported by respondents and learners in the level 5 patient records questionnaire.

Multimedia Appendix 8 Willingness to change practice reported in the level 3 to 4 and level 5 outcomes questionnaires.

Multimedia Appendix 9 Unmet educational needs identified by learners.

## Abbreviations

CME: continuing medical education
DCES: diabetes care and education specialist
DPP-4: dipeptidyl peptidase-4
GIP: glucose-dependent insulinotropic polypeptide
GLP-1: glucagon-like peptide-1
HbA1c: hemoglobin A1c
HCP: health care professional
MDT: multidisciplinary team
RA: receptor agonist
SGLT2i: sodium-glucose cotransporter-2 inhibitor
T2D: type 2 diabetes

## Data Availability

Data will be shared with bona fide researchers submitting a research proposal to touch Independent Medical Education. Access requests should be submitted to AN (Alex.Noble@touchime.org). Data will be made available from the study publication date. Any individual participant data will be shared in data sets in a deidentified and anonymized format.
