# Supplementary material for: Impact on Knowledge, Competence, and Performance of a Faculty-Led Web-Based Educational Activity for Type 2 Diabetes and Obesity: Questionnaire Study Among Health Care Professionals and Analysis of Anonymized Patient Records
Source: JMIR Form Res. 2023 Sep 13;7:e49115. doi: 10.2196/49115 (PMC10534284; doi:10.2196/49115)
Supplement: Multimedia Appendix 1 [file formative_v7i1e49115_app1.docx]

**Multimedia Appendix 1: Supplementary Methods**

**Activity development**

The educational activity was a touchMDT titled, “The future for glycemic control and weight loss in type 2 diabetes (T2D) and obesity: Incretin-based dual-agonists and optimizing patient education”. The learning objectives were: (1) to recognize the effect of incretin hormones and glucagon on metabolism in wellness and in T2D, (2) to evaluate the rationale and latest evidence for incretin-based dual-agonist therapies in patients with T2D and obesity, and (3) to apply early treatment intensification, weight loss, and patient education strategies in patients with T2D and obesity. The activity comprised three 10-15-minute videos (providing 37 minutes of education in total) and involved multidisciplinary team (MDT) members (an endocrinologist specializing in diabetes, an endocrinologist specializing in obesity, and a diabetes care and education specialist [DCES]) discussing their role in the management of patients with T2D and obesity with a patient with T2D.

**Communication channels**

Communication channels used to reach the target audience included direct publicity emails to the touchENDOCRINOLOGY database within the first 12 weeks of the activity launch, with a further reminder at around 6 months, display banners on the touchENDOCRINOLOGY website, advertisements in the peer-reviewed journal *US Endocrinology,* publicity via various relevant medical society partnerships, and HCP-targeted social media partnerships on Facebook, LinkedIn, and Twitter throughout the lifetime of the activity.

**Assessment of participation (level 1) and satisfaction (level 2)**

Moore’s level 1 (participation) was assessed over the first 6 months after launch. Both the number of healthcare professionals (HCPs) who engaged in the activity and the average time spent viewing the videos were measured. Google Analytics captured geo-location, participant numbers, and the overall average time HCPs had spent on the activity. The level 2 questionnaire assessed satisfaction with the activity, and included the following 6 statements that were to be scored using a 1-5 Likert scale (where 5 is the highest satisfaction): this activity was of high quality; this activity met the stated educational objectives; the content was free from commercial bias; the presenters were knowledgeable and effective; the activity contained content relevant to my clinical practice; and the information presented is likely the help change my management strategies in this therapeutic area.
