# Supplementary material for: Impact on Knowledge, Competence, and Performance of a Faculty-Led Web-Based Educational Activity for Type 2 Diabetes and Obesity: Questionnaire Study Among Health Care Professionals and Analysis of Anonymized Patient Records
Source: JMIR Form Res. 2023 Sep 13;7:e49115. doi: 10.2196/49115 (PMC10534284; doi:10.2196/49115)
Supplement: Multimedia Appendix 2 [file formative_v7i1e49115_app2.docx]

**Multimedia Appendix 2: Questions included in the level 3 and 4 outcomes questionnaire.**

| **Questions/multiple choice answers*** |
| --- |
| 1. Which statement best summarizes the action of the endogenous incretin hormones after food intake in healthy individuals?   A. They stimulate insulin and glucagon secretion, resulting in elevated blood glucose levels  B. They inhibit insulin and glucagon secretion, resulting in lowering of blood glucose levels  C. They stimulate insulin secretion and reduce insulin sensitivity, resulting in swings in blood glucose levels  D. **They stimulate insulin and inhibit glucagon secretion, resulting in lowering of blood glucose levels** |
| 1. Which of the following statements reflects findings from recent preclinical research on the mechanism of action of the dual GIP and GLP-1 receptor agonist, tirzepatide, in obese insulin-resistant mice?   A. **Insulin sensitivity was improved in a weight-dependent and -independent manner. GIPR agonism mediated the weight-independent sensitization**  B. Insulin sensitivity was improved in a weight-independent manner, mediated by GIPR antagonism  C. Insulin sensitivity was improved in a weight-dependent**—**but not independent **—**manner, mediated by GIPR agonism  D. Insulin sensitivity was reduced in a weight-dependent manner, via GIPR agonism |
| 1. Incretin-based dual GIP and GLP-1 receptor agonists may offer an additional treatment option for patients with type 2 diabetes and obesity in the future. Based on the results of the SURPASS clinical trial program, if approved, how would you communicate to your patients on the potential benefits of these agents after 9-12 months of treatment?   A. They can reduce HbA1c levels by ~0.5%-point and reduce body weight by up to 10%  B. They can reduce HbA1c levels by ~1%-point and reduce body weight by up to 5%  C. **They can reduce HbA1c levels by ~2%-point and reduce body weight by up to 14%**  D. They can reduce HbA1c levels by ~5%-point and reduce body weight by up to 28% |
| 1. Cotadutide, a dual GLP-1 and glucagon receptor agonist, has been investigated in a phase IIb study in patients with type 2 diabetes and overweight inadequately controlled by metformin. Which statement best describes the % change in body weight with cotadutide versus placebo at 14 weeks?   A. **Up to -5% with cotadutide versus –0.7% with placebo**  B. Up to -5% with cotadutide versus +2.7% with placebo  C. Up to -10% with cotadutide versus +0.7% with placebo  D. Up to -15% with cotadutide versus –2.7% with placebo |
| 1. Your 50-year-old female patient with type 2 diabetes and obesity has not met her treatment targets. After 3 months of metformin therapy, her HbA1c is 9% and her BMI remains at 33 kg/m^2^. What is your rationale for early intensification of therapy beyond metformin?   A. To rapidly reduce β-cell function prior to transitioning to insulin therapy  B. To prevent acquired resistance to metformin therapy  C. To improve glycemic control and keep weight stable  D. **To improve glycemic control and avoid long-term complications of type 2 diabetes** |
| 1. Your patient from the previous question (50-year-old female; type 2 diabetes & obesity; HbA1c 9% after 3 months of metformin therapy) also has established ASCVD. What add-on therapy would you choose to intensify her treatment regimen?   A. A basal insulin  B. A sulfonylurea  C. **A GLP-1 RA or SGLT2 inhibitor**  D. A DPP-4 inhibitor |
| 1. Your 48-year-old female patient with type 2 diabetes is showing signs of non-adherence to her medication and tells you that she finds her injection device difficult to use. What steps would you take to address this?   A. Provide her with an online resource that instructs patients on how to inject  B. Provide ongoing, individualized diabetes education  C. Arrange an appointment for her with a diabetes nurse and follow-up at 18 months  D. Switch her to a non-injectable therapy |

*The correct answer is indicated in bold. Level 3 questions were structured to assess knowledge of guidelines and clinical trial data and how these may be applied in clinical practice, whereas level 4 questions were structured as patient cases to directly assess competence in making the optimal clinical decision. Respondents and learners are defined as healthcare professionals who completed the pre- and postactivity questionnaires, respectively.

**Abbreviations:** ASCVD, atherosclerotic cardiovascular disease; DPP-4, dipeptidyl peptidase-4; GIP, glucose-dependent insulinotropic polypeptide; GIPR, glucose-dependent insulinotropic polypeptide receptor; GLP-1, glucagon-like peptide-1; GLP-1 RA, glucagon-like peptide-1 receptor agonist; HbA1c, glycated hemoglobin; SGLT2, sodium-glucose cotransporter-2.
