# Supplementary material for: Impact on Knowledge, Competence, and Performance of a Faculty-Led Web-Based Educational Activity for Type 2 Diabetes and Obesity: Questionnaire Study Among Health Care Professionals and Analysis of Anonymized Patient Records
Source: JMIR Form Res. 2023 Sep 13;7:e49115. doi: 10.2196/49115 (PMC10534284; doi:10.2196/49115)
Supplement: Multimedia Appendix 3 [file formative_v7i1e49115_app3.docx]

**Multimedia Appendix 3: Questions included in the level 5 outcomes questionnaire.**

| **Questions/multiple choice answers*** |
| --- |
| 1. What class of second-line treatment do you select for your patients treated with metformin who have poorly controlled T2D and obesity, without established ASCVD or CKD, and who wish to maximize weight loss?   A. TZD or SU  B. DPP-4 inhibitor  C. **GLP-1 RA or SGLT2 inhibitor**  D. Basal insulin |
| 1. Your patient with T2D and obesity is starting second-line treatment with a GLP-1 RA. What important side effects do you advise your patient to expect while on treatment?   A. Diabetic retinopathy  B. **Gastrointestinal effects, including nausea**  C. Pancreatitis  D. Modest increase in heart rate |
| 1. What management approach do you use for your patients with long-standing T2D and obesity who are on dual therapy, but whose HbA1c levels remain above their individualized target?   A. **Further intensify treatment (triple therapy)**  B. Switch to a different second-line therapy  C. Continue on the current regimen, with monitoring at 3-6 monthly intervals |
| 1. What approach do you implement to educate your patients with T2D and obesity on how to manage their diabetes and prevent/delay complications?   A. Patient self-education using online diabetes resources  B. Physician-led education during a clinic visit around the time of diagnosis  C. **Ongoing multidisciplinary team approach, including a diabetes educator**  D. No specific diabetic education or training beyond patient leaflets |

*The best clinical option is indicated in bold.

**Abbreviations:** ASCVD, atherosclerotic cardiovascular disease; CKD, chronic kidney disease; DPP-4, dipeptidyl peptidase-4; GLP-1 RA, glucagon-like peptide-1 receptor agonist; HbA1c, glycated hemoglobin; SGLT2, sodium-glucose cotransporter-2; SU, sulfonylurea; T2D, type 2 diabetes; TZD, thiazolidinedione.
