# Supplementary material for: Impact on Knowledge, Competence, and Performance of a Faculty-Led Web-Based Educational Activity for Type 2 Diabetes and Obesity: Questionnaire Study Among Health Care Professionals and Analysis of Anonymized Patient Records
Source: JMIR Form Res. 2023 Sep 13;7:e49115. doi: 10.2196/49115 (PMC10534284; doi:10.2196/49115)
Supplement: Multimedia Appendix 4 [file formative_v7i1e49115_app4.docx]

**Multimedia Appendix 4: Summary of correct responses for the level 3 to 4 outcomes questionnaire before and after the launch of touchMDT by level of experience of the respondents and learners.**

The box and whisker plot shows the distribution of the number of correctly answered questions by all respondents and learners. The horizontal red line within the box indicates the median, the “x” symbol represents the mean, the boxes indicate the IQR, and the vertical lines (whiskers) extend to the range of values, excluding outliers. Outliers are defined as values that fall outside a distance of 1.5× the IQR from the upper and lower quartiles and are represented by empty circles. Respondents and learners are defined as healthcare professionals who completed the pre- and postactivity questionnaires, respectively. *P* values compare the difference between pre- and postactivity scores^a^ and between subgroups^b^.

**
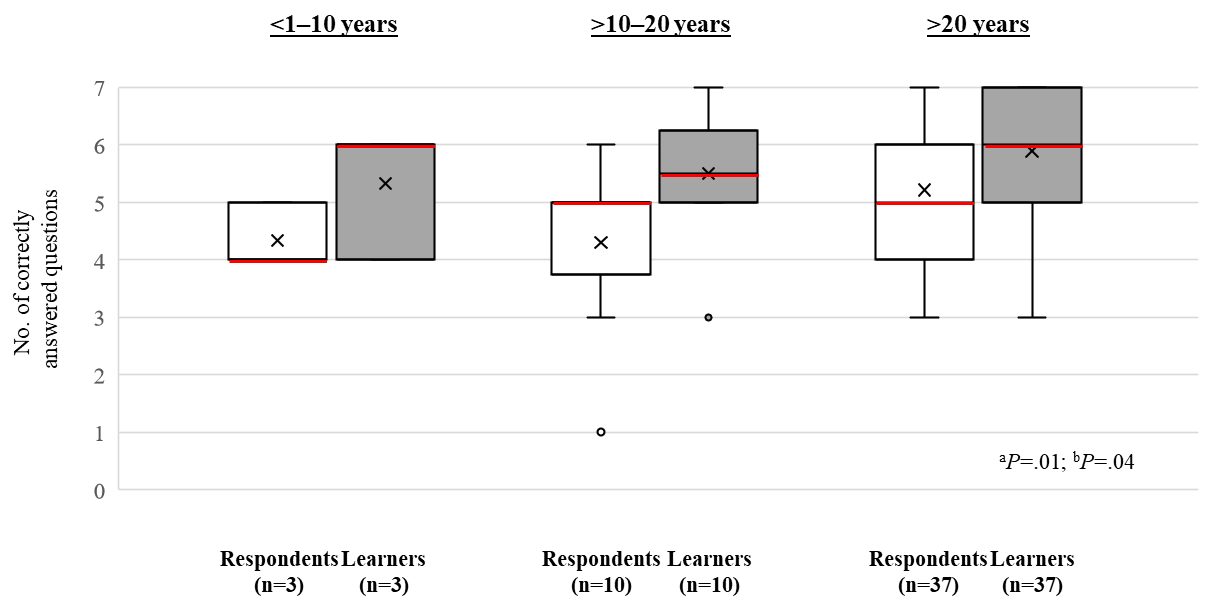
**
