# Supplementary material for: Impact on Knowledge, Competence, and Performance of a Faculty-Led Web-Based Educational Activity for Type 2 Diabetes and Obesity: Questionnaire Study Among Health Care Professionals and Analysis of Anonymized Patient Records
Source: JMIR Form Res. 2023 Sep 13;7:e49115. doi: 10.2196/49115 (PMC10534284; doi:10.2196/49115)
Supplement: Multimedia Appendix 5 [file formative_v7i1e49115_app5.docx]

**Multimedia Appendix 5: Baseline characteristics for patients in the level 5 patient records questionnaire.**

|  | **Respondents**  **n=50** | **Learners**  **n=50** |
| --- | --- | --- |
| **Age, years, mean (SD)** | 53.7 (10.4) | 52.5 (11.3) |
| **Race, n (%)** |  |  |
| **Caucasian** | 42 (84) | 37 (74) |
| **Black/African American** | 4 (8) | 7 (14) |
| **Asian** | 3 (6) | 3 (6) |
| **Other** | 1 (2) | 3 (6) |
| **BMI, kg/m^2^, mean (SD)** | 32.3 (4.5) | 32.6 (3.7) |
| **Non-smokers, n (%)** | 48 (96) | 47 (94) |
| **Health insurance, n (%)** |  |  |
| **Medicaid** | 5 (10) | 3 (6) |
| **Preferred provider organization** | 23 (46) | 18 (36) |
| **Exclusive provider organization** | 8 (16) | 4 (8) |
| **Health maintenance organization** | 6 (12) | 18 (36) |
| **Medicare** | 7 (14) | 6 (12) |
| **Other** | 1 (2) | 1 (2) |
