# Supplementary material for: Impact on Knowledge, Competence, and Performance of a Faculty-Led Web-Based Educational Activity for Type 2 Diabetes and Obesity: Questionnaire Study Among Health Care Professionals and Analysis of Anonymized Patient Records
Source: JMIR Form Res. 2023 Sep 13;7:e49115. doi: 10.2196/49115 (PMC10534284; doi:10.2196/49115)
Supplement: Multimedia Appendix 6 [file formative_v7i1e49115_app6.docx]

**Multimedia Appendix 6: Additions or changes to diabetes treatment reported by respondents and learners in the level 5 patient records questionnaire.**

Table shows the number of patients who added or switched to a diabetes treatment at their most recent visit. Respondents and learners are defined as healthcare professionals who completed the pre- and postactivity questionnaires, respectively.

| **Patients with an addition or change in diabetes treatment at the most recent visit, n (%)** | **Respondents**  **n=50** | **Learners**  **n=50** |
| --- | --- | --- |
| **Any addition or change** | **13 (26)** | **15 (30)** |
| **GLP-1 RA** | 10 (77) | 9 (60) |
| **SGLT2i** | 2 (15) | 2 (13) |
| **Insulin (adjusted dose)** | 1 (8) | 0 |
| **Basal insulin** | 0 | 2 (13) |
| **GLP-1 RA (adjusted dose)** | 0 | 1 (7) |
| **Dual GIP/GLP-1 RA** | 0 | 1 (7) |

**Abbreviations:** GIP, glucose-dependent insulinotropic polypeptide; GLP-1, glucagon-like peptide-1; RA, receptor agonist; SGLT2, sodium-glucose cotransporter-2.
