# Supplementary material for: Impact on Knowledge, Competence, and Performance of a Faculty-Led Web-Based Educational Activity for Type 2 Diabetes and Obesity: Questionnaire Study Among Health Care Professionals and Analysis of Anonymized Patient Records
Source: JMIR Form Res. 2023 Sep 13;7:e49115. doi: 10.2196/49115 (PMC10534284; doi:10.2196/49115)
Supplement: Multimedia Appendix 7 [file formative_v7i1e49115_app7.docx]

**Multimedia Appendix 7: Reasons given for addition or changes to diabetes treatment reported by respondents and learners in the level 5 patient records questionnaire.**

The table shows the reasons given for the addition or change in diabetes treatment at the most recent visit. Respondents and learners are defined as healthcare professionals who completed the pre- and postactivity questionnaires, respectively.

| **Reason given for an addition or change in diabetes treatment at the most recent visit, n/N (%)** | | | | | |
| --- | --- | --- | --- | --- | --- |
|  | **Efficacy (improved glycemic control)** | **Efficacy  (weight loss)** | **Efficacy (cardiorenal outcomes)** | **Low risk of hypoglycemia** | **Favorable side effect profile** |
| **Respondents** | | | | | |
| **GLP-1 RA** | 9/10 (90) | 10/10 (100) | 7/10 (70) | 5/10 (50) | 1/10 (10) |
| **SGLT2i** | 2/2 (100) | 2/2 (100) | 1/2 (50) | 0 | 0 |
| **Insulin (adjusted dose)** | 1/1 (100) | 0 | 0 | 0 | 0 |
| **Learners** | | | | | |
| **GLP-1 RA** | 7/9 (8) | 9/9 (100) | 6/9 (7) | 3/9 (33) | 1/9 (1) |
| **SGLT2i** | 2/2 (100) | 1/2 (50) | 1/2 (50) | 1/2 (50) | 0 |
| **Basal insulin** | 2/2 (100) | 0 | 0 | 0 | 0 |
| **GLP-1 RA (adjusted dose)** | 1/1 (100) | 1/1 (100) | 0 | 0 | 0 |
| **Dual GIP/GLP-1 RA** | 1/1 (100) | 1/1 (100) | 0 | 0 | 0 |

**Abbreviations:** GIP, glucose-dependent insulinotropic polypeptide; GLP-1, glucagon-like peptide-1; n, number of patients with the reason given; N, number of patients who added or switched to the specific diabetes treatment; RA, receptor agonist; SGLT2, sodium-glucose cotransporter-2.
