# Supplementary material for: Impact on Knowledge, Competence, and Performance of a Faculty-Led Web-Based Educational Activity for Type 2 Diabetes and Obesity: Questionnaire Study Among Health Care Professionals and Analysis of Anonymized Patient Records
Source: JMIR Form Res. 2023 Sep 13;7:e49115. doi: 10.2196/49115 (PMC10534284; doi:10.2196/49115)
Supplement: Multimedia Appendix 8 [file formative_v7i1e49115_app8.docx]

**Multimedia Appendix 8:** **Willingness to change practice reported in the level 3 to 4 and level 5 outcomes questionnaires.**

Table shows the responses of learners to the question, “As a result of your participation in this session, will you make a change in your practice?”

|  | **Level 3 to 4 (N=50)** | **Level 5 (N=50)** |
| --- | --- | --- |
| **Yes, n (%)** | 31 (62) | 29 (58) |
| **Uncertain, more education needed, n (%)** | 4 (8) | 2 (4) |
| **Uncertain, practical limitations, n (%)** | 7 (14) | 6 (12) |
| **No, more education needed, n (%)** | 2 (4) | 2 (4) |
| **No, practical limitations, n (%)** | 6 (12) | 11 (22) |
