# Supplementary material for: Impact on Knowledge, Competence, and Performance of a Faculty-Led Web-Based Educational Activity for Type 2 Diabetes and Obesity: Questionnaire Study Among Health Care Professionals and Analysis of Anonymized Patient Records
Source: JMIR Form Res. 2023 Sep 13;7:e49115. doi: 10.2196/49115 (PMC10534284; doi:10.2196/49115)
Supplement: Multimedia Appendix 9 [file formative_v7i1e49115_app9.docx]

**Multimedia Appendix 9: Unmet educational needs identified by learners.**

The top three unmet educational needs are shown, as identified by learners who completed the level 3 to level 4 and level 5 questionnaires following launch of the touchMDT activity. Learners were required to rank four, predefined, potential educational gaps in response to the question, “What do you think is the most important unmet educational need in this therapy area?”

| **Level 3 to 4 questionnaire** | **Level 5 questionnaire** |
| --- | --- |
| 1. Efficacy and safety data for incretin-based dual agonists and their future role in clinical practice | 1. Understanding the biology and psychology of obesity and approaches for its prevention and management |
| 2. Understanding the biology and psychology of obesity and approaches for its prevention and management | 2. The role of diabetes care and education specialists in the T2D and obesity MDT |
| 3. The role of diabetes care and education specialists in the T2D and obesity MDT | 3. Efficacy and safety data for incretin-based dual agonists and their future role in clinical practice |

## 
